# Data-driven autonomous operation of VOCs removal system

**DOI:** 10.1038/s41598-024-56502-7

**Published:** 2024-03-12

**Authors:** Myeonginn Kang, Jongmin Han, Yangjoon Kim, Seongcheon Kim, Seokho Kang

**Affiliations:** 1 Department of Industrial Engineering, Sungkyunkwan University, Jangan-gu, Suwon, 16419 Republic of Korea; 2 Shinsung E&G Co. Ltd., Gwacheon, 13840 Republic of Korea

**Keywords:** Environmental chemistry, Computer science

## Abstract

Removal of volatile organic compounds (VOCs) from the air has been an important issue in many industrial fields. Traditionally, the operation of VOCs removal systems has relied on fixed operating conditions determined by domain experts based on their expertise and intuition. In practice, this manual operation cannot respond immediately to changes in the system environment. To facilitate the autonomous operation of the system, the operating conditions should be optimized properly in real time to adapt to the changes in the system environment. Recently, optimization frameworks have been widely applied to real-world industrial systems across various domains using different approaches. The primary motivation for this study is the effective implementation of an optimization framework targeting a VOCs removal system. In this paper, we present a data-driven autonomous operation method for optimizing the operating conditions of a VOCs removal system to enhance the overall performance. An optimization problem is formulated with the decision variables denoting the parameters associated with the operating condition, the environmental variables representing the measurements for the system environment, the constraints specifying the control ranges of the parameters, and the objective function representing the system performance as determined by the operating conditions and environment. Using the previous operation data from the system, a neural network is trained to model the system performance as a function of the decision and environmental variables to approximate the objective function. For the current state of the system environment, the optimal operating condition is derived by solving the optimization problem. A case study of a targeted VOCs removal system demonstrates that the proposed method effectively optimizes the operating conditions for improved system performance without intervention from domain experts.

## Introduction

Volatile organic compounds (VOCs) are a group of chemicals that easily evaporate into the air at room temperature and pressure. They are the major air pollutants in many industrial fields, such as furniture manufacturing, vehicle manufacturing, printing, and equipment coating^1^. VOCs participate in atmospheric photochemical oxidation, which is harmful to the environment^2^. Owing to the well-known hazards associated with VOCs^3^ the effective treatment of VOCs in the air has been an important industrial issue with ongoing technological challenges^4^.

To control the emission of VOCs, a VOCs removal system uses chemical techniques, which can be mainly categorized into recovery and destruction approaches^5–7^. The recovery approach changes the temperature and pressure to separate VOCs using techniques such as absorption^8^, adsorption^9,10^, membrane separation^11^, and condensation^12^. The destruction approach decomposes VOCs into carbon dioxide, water, and non-toxic or less toxic compounds using techniques such as thermal^13^, biological^14,15^, and catalytic oxidation^16–19^.

**Figure 1:**
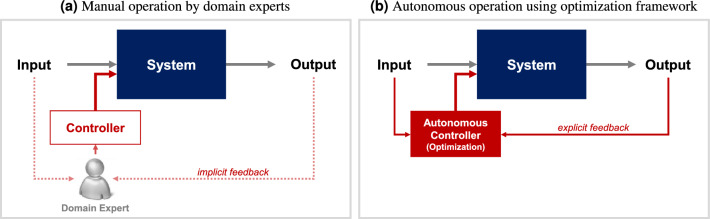
Approaches to operating VOCs removal system.

Traditionally, VOCs removal systems have been manually operated using fixed operating conditions determined by domain experts, as shown in Fig. 1a. This manual operation requires an in-depth understanding of the mechanism of the system, which may be difficult because of the complex relationship between the operating conditions, system environment, and system performance. Thus, the system performance depends significantly on the expertise and intuition of domain experts. Moreover, in practice, the system environment changes gradually over time. Thus, the operating conditions should be dynamically adjusted to adapt to the changes, which is difficult to perform manually.

In many industrial fields, extensive research efforts have been directed to introduce optimization frameworks for automating system operations. The aim is to automatically optimize the operating conditions to improve the system performance. An optimization problem is formulated by defining the decision variables to be optimized, identifying the constraints imposed on the decision variables, defining the environmental variables representing the system environment, and designing the objective function to be minimized. The general formulation can be mathematically described as follows:

1 $$\begin{aligned} \begin{aligned}{}\underset{\textbf{x}}{\text {minimize}}\quad{} & {} {J}(\textbf{x},\textbf{z})\\\text {subject to}\quad{} & {} g_i(\textbf{x}) \le 0,\quad i=1,\ldots ,m;\\{} {} {}&h_j(\textbf{x}) = 0,\quad j=1,\ldots ,n. \end{aligned} \end{aligned}$$

The decision variables $\textbf{x}$ are set as the parameters associated with the operating conditions of the system. The environmental variables $\textbf{z}$ are externally measured or determined from the system environment. The constraints $g_i(\textbf{x}) \le 0$ and $h_j(\textbf{x}) = 0$ indicate the control ranges of the parameters. The objective function *J* reflects the objectives of the system operation, which relies on the decision and environmental variables. The optimal operating condition is derived by solving the optimization problem to find the values of the decision variables that minimize the objective function while satisfying the constraints.

Optimization frameworks have been widely applied in the design and operation of real-world systems in various domains, including recovery processes^20,21^, machining processes^22^, photovoltaic systems^23^, HVAC systems^24,25^, material designs^26,27^, and circuit designs^28–30^. These studies formulated their optimization problems in different ways to optimize the operating conditions of the target system. They can be categorized based on how the objective function is designed and how the optimization problem is solved. Regarding the design of the objective function, three approaches have mainly been presented to approximate the outcome of the operation of a real system:

- *Hand-crafted function*: The objective function is manually designed as a linear or quadratic function based on the knowledge and intuition of domain experts^31,32^. This approach is applicable if the target system exhibits a simple and well-understood mechanism. However, hand-crafting the objective function is difficult in practice if the target system is complex and the domain experts lack sufficient knowledge.
- *Simulation*: This approach uses a simulation model that imitates the operation of the target system^20,21,23,24,26,28–30,33^. The objective function is evaluated by virtually running the target system through simulation. Despite its effectiveness, simulation models may not be available for use in many real-world systems. In addition, this approach is generally computationally expensive and time-consuming compared with other approaches.
- *Machine learning*: A data-driven prediction model is built by learning from the previous operational data collected from the target system^22,25,27^. Various learning algorithms are readily usable, such as linear regression, random forests, support vector machines, and neural networks. The effectiveness of this approach depends significantly on the quantity and quality of the data.

Depends on the type of the objective function, four approaches have been mainly used to solve the optimization problems:

- *Linear/quadratic programming*: They are traditional approaches for solving specific types of optimization problems, wherein a linear or quadratic objective function is minimized subject to linear constraints on the decision variables^31,32^.
- *Meta-heuristics*: This approach uses stochastic components to efficiently explore the search space to find near-optimal solutions for optimization problems that are difficult to solve using traditional approaches^20–25,33^. Representative algorithms include particle swarm optimization, simulated annealing, and evolutionary and genetic algorithms. They make relatively few assumptions regarding the optimization problem and are applicable to any type of objective function.
- *Bayesian optimization*: This approach attempts to efficiently optimize an expensive-to-evaluate objective function using a small number of function evaluations^26,29,30^. A surrogate model is fit to previous function evaluations. The stochastic predictions by the surrogate model are used to decide where to evaluate next in updating the surrogate model. No assumptions are made about the objective function.
- *Gradient-based optimization*: This approach iteratively searches for the directions defined by the gradient of the objective function to solve the problem^27^. This approach is applicable when the objective function is differentiable with respect to the decision variables. Examples include gradient descent and quasi-Newton methods.

This study aims to implement a data-driven autonomous operation method for a VOCs removal system, as schematically illustrated in Fig. 1b. The VOCs removal system targeted in this study, CV-Master, is developed by Shinsung E&G and employs adsorption and catalytic oxidation techniques for the VOCs removal process. We formulate an optimization problem by identifying the decision, environmental, and dependent variables of the system, the constraints for the decision variables, and the objective function that reflects the objectives of the system operation. The main challenge lies in mathematically expressing how the system is intended to operate as a function of the decision and environmental variables. Due to the complexity and incomplete understanding of the target system, a hand-crafted objective function may not precisely reflect the actual objective. Additionally, there is no available simulation model that emulates the target system. To address this difficulty, we adopt a machine learning approach, which has proven effective in optimizing operating conditions for complex systems. With expressing the dependent variables $\textbf{y}$, the objective function $\tilde{{J}}$ can be designed in a simpler form. The prediction model *f* predicts the dependent variables $\textbf{y}$ as a function of the decision and environmental variables, i.e., $\hat{\textbf{y}}=f(\textbf{x},\textbf{z})$. The predicted dependent variables $\hat{\textbf{y}}$ are then used to approximately express the objective function $\tilde{{J}}$. The modified formulation can be described as follows:

2 $$\begin{aligned} \begin{aligned}{}\underset{\textbf{x}}{\text {minimize}}\quad{} & {} \tilde{{J}}(\textbf{x},\textbf{z},\hat{\textbf{y}})\\\text {subject to}\quad{} & {} \hat{\textbf{y}}=f(\textbf{x},\textbf{z}); \\{} {} {}&g_i(\textbf{x}) \le 0,\quad i=1,\ldots ,m;\\{} {} {}&h_j(\textbf{x}) = 0,\quad j=1,\ldots ,n. \end{aligned} \end{aligned}$$

For the prediction model *f*, a neural network is trained by learning the working mechanism of the system from the previous operational data collected from the system. The neural network is used to approximately represent the objective function $\tilde{{J}}$ in a differentiable form. Given the state of the system environment, the operating condition is optimized by solving the optimization problem to improve the system performance. Because the approximate objective function is differentiable, the optimization problem is solved using a gradient-based optimization algorithm. We investigate the effectiveness of the proposed method in optimizing the actual operation of the target system.

## Method

### VOCs removal system

**Figure 2:**
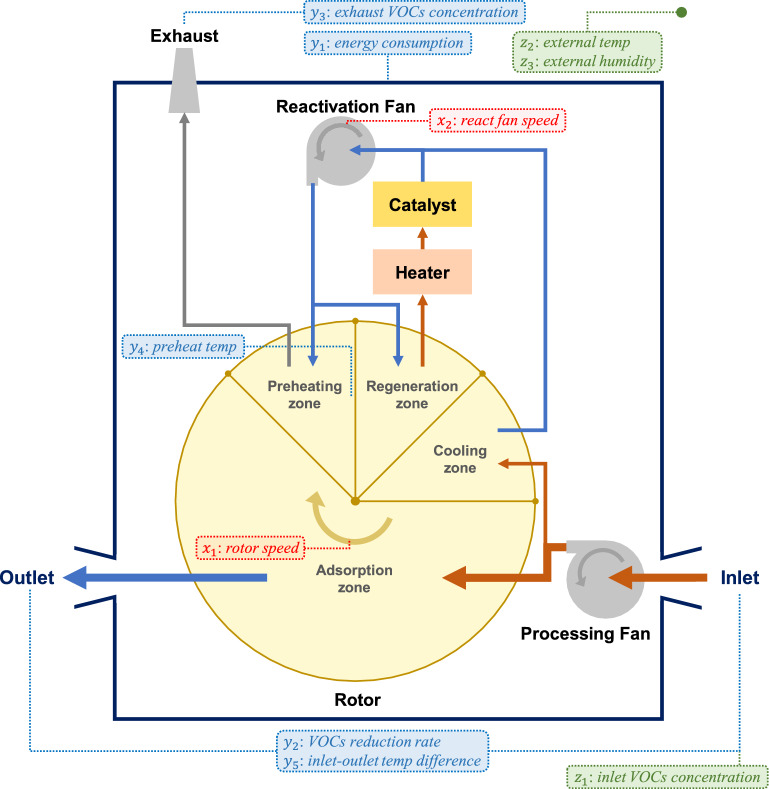
Schematic illustration for VOCs removal process of the target system.

**Table 1:** Description of variables in the target system.

Type	Variable	Name	Description	Control range/objective	Unit
Decision variable	$x_1$	Rotor speed	Rotational speed of the rotor	[2, 4.4]	RPH
	$x_2$	React fan speed	Rotational speed of the reactivation fan	[30, 55]	Hz
Environmental variable	$z_1$	Inlet VOCs concentration	VOCs concentration in the inlet air	–	ppm
	$z_2$	External temp	Temperature of the external air	–	^∘^C
	$z_3$	External humidity	Relative humidity of the external air	–	$\%$
Dependent variable	$y_1$	Energy consumption	Electronic energy consumed by the system operation	Minimize	kW
	$y_2$	VOCs reduction rate	Reduction rate of VOCs concentration from the inlet to outlet air	$\ge$95	$\%$
	$y_3$	Exhaust VOCs concentration	VOCs concentration in the exhaust	$\le$5	ppm
	$y_4$	Preheat temp	Temperature when transitioning from preheating to regeneration zone	$\le$220	^∘^C
	$y_5$	Inlet-outlet temp difference	Temperature difference between the outlet and inlet air	$\le$4	^∘^C

Figure 2 illustrates the target VOCs removal system developed by Shinsung E&G, named CV-Master. It uses a large circular ceramic rotor consisting of four zones: adsorption, preheating, regeneration, and cooling zones. The rotor is continuously rotated to alternately perform adsorption and regeneration to remove VOCs from the air. The external air with a high VOCs concentration first passes through the adsorption zone of the rotor. In the adsorption zone, the VOCs in the air are adsorbed on the rotor. The air with reduced VOCs concentration is emitted outside the system. The preheating zone increases the air temperature. In the regeneration zone, the VOCs are desorbed from the rotor at high temperatures and are subsequently decomposed into water and carbon dioxide via a catalytic oxidation reaction. The heat generated during the reaction is used in the preheating and regeneration zones to save energy. The cooling zone reduces the air temperature to facilitate the adsorption of the VOCs in the adsorption zone.

The decision, environmental, and dependent variables used in the optimization problem are listed in Table 1. Domain experts at Shinsung E&G have specified the variables associated with the CV-Master’s operation and monitoring. The decision variables (*rotor speed* and *react fan speed*) correspond to the parameters that determine the operating conditions of the system. The values of the decision variables can be adjusted within their respective control ranges to operate the system more efficiently. The environmental variables (*inlet VOCs concentration*, *external temp*, and *external humidity*) are external factors that affect the system performance. If the values of the environmental variables change, the operating conditions must be optimized to adapt to these changes. The dependent variables (*energy consumption*, *VOCs reduction rate*, *exhaust VOCs concentration*, *preheat temp*, and *inlet-outlet temp difference*) are observed as the outcomes of the system operation, depending on the decision and environmental variables. The performance of the system is monitored by measuring the values of the dependent variables. We denote the vectors of the decision, environmental, and dependent variables by $\textbf{x}=[x_1, x_2]$, $\textbf{z}=[z_1, z_2, z_3]$, and $\textbf{y}=[y_1, y_2, y_3, y_4, y_5]$, respectively.

The operational goal of the target system is to achieve a VOCs reduction rate of over 95% with lower energy consumption while satisfying the required constraints.

### Optimization problem

For the current state of the decision variables $\textbf{x}^0=[{x}_1^0,{x}_2^0]$ and environmental variables $\textbf{z}^{0}=[z_1^{0}, z_2^{0}, z_3^{0}]$, the goal is to optimize the values of decision variables, $x_1$ and $x_2$, to achieve the objectives while satisfying the required constraints for the operation of the target system, as listed in Table 1.

The objective function $\tilde{{J}}$ is designed to reflect the multiple objectives of the system operation in the form of a function of $\textbf{x}$, $\textbf{z}$, and $\textbf{y}$ as follows:

3 $$\begin{aligned} \begin{aligned} \tilde{{J}}(\textbf{x},\textbf{z},\textbf{y}) =&\text { }y_1 \\&+\alpha _2 \cdot \max (0, 95-y_2)^2 \\&+\alpha _3 \cdot \max (0, y_3 -5)^2 \\&+\alpha _4 \cdot \max (0, y_4 -220)^2 \\&+\alpha _5 \cdot \max (0, y_5-4)^2 \\&+ \beta _1 \cdot (x_1-x_1^{0})^2 + \beta _2 \cdot (x_2-x_2^{0})^2, \end{aligned} \end{aligned}$$

where $\alpha$ and $\beta$ are the weight hyperparameters for controlling the relative strengths of the individual objectives. The first term is for minimizing the energy consumption of the system. The second-to-fifth terms penalize failure to meet the target ranges of the corresponding dependent variables. The last two terms correspond to the minimization of the difference between the initial and optimized values of the decision variables, which helps prevent drastic changes in the operating conditions for the stability of system operation.

As the dependent variables cannot be instantly observed, they have to be predicted during the evaluation of the objective function for optimization. The prediction model *f* is used to predict the dependent variables as a function of the decision and environmental variables. Using the prediction model *f*, the predicted dependent variables $\hat{\textbf{y}}$ are obtained as follows:

4 $$\begin{aligned} \hat{\textbf{y}} = [\hat{y}_1, \hat{y}_2, \hat{y}_3, \hat{y}_4, \hat{y}_5] = f(\textbf{x}, \textbf{z}). \end{aligned}$$

The decision variables are constrained to satisfy the corresponding control ranges. The control ranges assigned to $x_1$ and $x_2$ are [2, 4.4] and [30, 55], respectively.

After the specification of the objective function $\tilde{{J}}$ and a set of constraints, the optimization problem is mathematically formulated as follows:

5 $$\begin{aligned} \begin{aligned}{}\underset{\textbf{x}}{\text {minimize}}\quad{} & {} \tilde{{J}}(\textbf{x},\textbf{z}^0,\hat{\textbf{y}}) \\\text {subject to}\quad{} & {} \hat{\textbf{y}}=f(\textbf{x},\textbf{z}^0); \\{} {} {}&2 \le x_1 \le 4.4;\\{} {} {}&30 \le x_2 \le 55. \end{aligned} \end{aligned}$$

Given $\textbf{x}^0$ and $\textbf{z}^0$, the optimal values of the decision variables, denoted by $\textbf{x}^*$, are found by solving the optimization problem. We adopt a gradient-based optimization approach. The solution $\textbf{x}^*$ is interpreted as the optimized operating condition of the system. It is important to note that the quality of the solution depends significantly on the predictive performance of the prediction model *f*.

**Figure 3:**
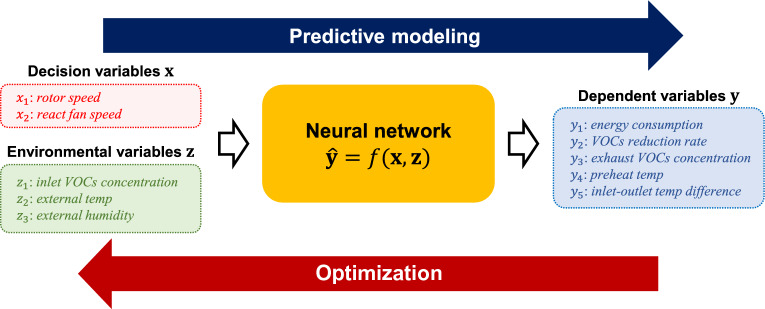
Schematic illustration of data-driven autonomous operation framework for the target system.

Figure 3 schematically shows the data-driven autonomous operation framework for the target system. The following subsections describe how to build the prediction model *f* and solve the optimization problem.

### Neural network approximation of objective function

Owing to the difficulty of observing the actual dependent variables $\textbf{y}$ directly during the optimization, the objective function $\tilde{{J}}$ is approximately evaluated using the predicted dependent variables $\hat{\textbf{y}}$ instead of the actual dependent variables $\textbf{y}$. We adopt a machine learning approach to build the prediction model *f* for predicting the dependent variables $\textbf{y}$ as a function of the decision variables $\textbf{x}$ and environmental variables $\textbf{z}$. This approach involves training the prediction model *f* using the previous operational data collected from the target system, represented as ${D}=\{(\textbf{x}_t,\textbf{z}_t,\textbf{y}_t)\}_{t=1}^T$, where $\textbf{x}_t$, $\textbf{z}_t$, and $\textbf{y}_t$ denote the observed values of variables for the *t*-th data instance. The trained model can then be used to predict the unknown value of the dependent variables $\textbf{y}_*$ for a query instance $(\textbf{x}_*,\textbf{z}_*)$, thereby enabling the estimation of the system performance under the new operating conditions and environment. Using the prediction model *f*, the objective function $\tilde{{J}}$ can be approximately expressed as a function of $\textbf{x}$ and $\textbf{z}$. The prediction model *f* must be sufficiently accurate to emulate the actual working mechanism of the system.

In this study, the prediction model *f* is built as a multi-output neural network that predicts all five dependent variables. Neural networks have demonstrated remarkable performance in various applications^34^ based on their ability to represent and learn the complex non-linear relationships between inputs and outputs without strong restrictions^35^. In addition, neural networks can be efficiently updated without forgetting its existing knowledge when new data becomes available^36^.

To model the empirical relationship between the decision, environmental, and dependent variables observed in the target system, the prediction model *f* learns the working mechanism of the system from a training dataset ${D}=\{(\textbf{x}_t,\textbf{z}_t,\textbf{y}_t)\}_{t=1}^T$, which comprises the previous operational records collected by the system. The model *f* is trained by minimizing the squared error loss ${L}(\hat{\textbf{y}}, \textbf{y}) = \Vert \hat{\textbf{y}} - {\textbf{y}} \Vert ^2$ on the training dataset *D*.

### Optimization algorithm

Since a neural network is a differentiable function, the objective function $\tilde{{J}}$, which is approximated with the predicted dependent variables $\hat{\textbf{y}}$, is differentiable with respect to the decision variables $\textbf{x}$. To efficiently solve the optimization problem in Eq. (5), we adopt a gradient-based optimization approach, known to be particularly useful when an objective function is expressed through a machine learning approach^37^.

We employ the limited-memory Broyden–Fletcher–Goldfarb–Shanno with bound constraints (L-BFGS-B) algorithm^38^, one of quasi-Newton methods, to perform optimization. L-BFGS-B is an extension of L-BFGS^39^ to handle simple bound constraints on the decision variables. This algorithm typically converges faster than other gradient-based optimization algorithms. In addition, it is memory-efficient and does not require careful configuration tuning.

The current state of the decision variables $\textbf{x}^0$ is used as the starting point for the optimization. Given $\textbf{z}=\textbf{z}^0$, the optimization proceeds by iteratively updating the values of the decision variables $\textbf{x}$ to find the local minimum of the objective function $\tilde{{J}}$ subject to the specified constraints. The solution $\textbf{x}^*$ corresponds to the values of the decision variables leading to the local minimum.

## Results and discussion

### Data description

We investigated the effectiveness of the proposed method by evaluating it in optimizing the operation of CV-Master, the VOCs removal system developed by a manufacturer. A dataset comprising 169 operational records under various operating conditions was collected from the target system. Each record contained the values of the decision, environmental, and dependent variables observed during data collection. Table 2 presents the descriptive statistics of the variables in the dataset.

**Table 2:** Descriptive statistics of variables in the dataset.

Type	Variable	Mean	Std. Dev.	Min	Max
Decision variable	$x_1$	3.00	0.62	1.10	4.40
	$x_2$	40.24	5.57	26.00	50.00
Environmental variable	$z_1$	15.23	6.52	1.45	26.11
	$z_2$	21.78	2.51	16.76	28.48
	$z_3$	29.34	9.42	15.06	44.83
Dependent variable	$y_1$	24.64	1.54	21.48	30.74
	$y_2$	95.78	1.97	90.79	99.17
	$y_3$	3.38	3.16	0.07	17.35
	$y_4$	202.79	14.10	160.67	230.67
	$y_5$	3.40	0.45	2.69	5.46

The variables were measured at different scales and thus had different ranges of values. To place all variables on the same scale, each variable was standardized to have zero mean and unit variance in the dataset.

### Performance evaluation of prediction models

For the prediction model *f*, we evaluated the predictive performance of neural networks with a hidden layer having 2, 3, 5, 10, and 20 hidden units, denoted by NN(2), NN(3), NN(5), NN(10), and NN(20), respectively. The tanh activation function was applied to the hidden units. We used the L-BFGS algorithm to train each neural network, during which L2 regularization was applied to the parameters. For reference, we also compared random forest (RF), ridge regression (Ridge), and k-nearest neighbors (k-NN) as baseline models. All models were implemented using the scikit-learn package, with the unspecified configurations set to the default in the package.

The predictive performance was evaluated using a ten-fold cross-validation procedure. In this procedure, the original dataset was split into ten folds. Each fold was then used once as a test set to measure the performance, with the remaining folds combined into a training set to train a prediction model. As the performance measures, we used root mean square error (RMSE) and coefficient of determination ($R^2$). Lower RMSE and higher $R^2$ values indicate better predictive performance.

**Figure 4:**
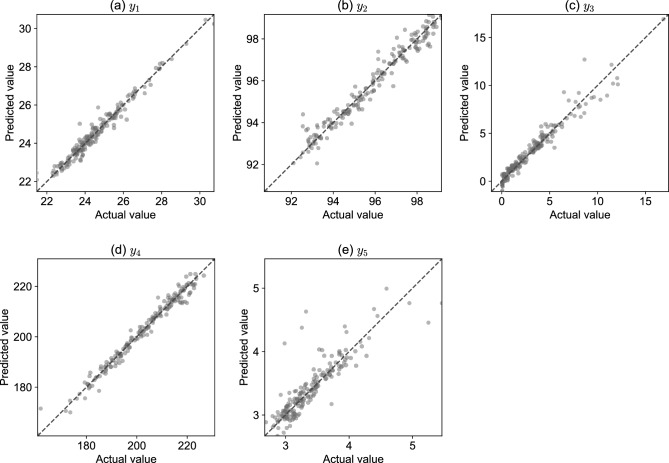
Relationship between actual and predicted values according to NN(10) for each dependent variable.

Table 3 presents the performance evaluation results of the compared models for the five dependent variables in terms of RMSE and $R^2$. The best RMSE and $R^2$ values for each dependent variable are represented in bold. NN(10) consistently yielded high validation performance for all dependent variables. Accordingly, we selected NN(10) to predict the dependent variables. Figure 4 presents scatter plots comparing the actual values to the predicted values using NN(10) for the dependent variables, visually demonstrating that the predicted values closely align with the actual values for each dependent variable.

**Table 3:** Comparison of the predictive performance of various prediction models (average ± standard deviation).

Dependent variable	Measure	NN(2)	NN(3)	NN(5)	NN(10)	NN(20)	RF	Ridge	k-NN
$y_1$	RMSE	0.8570 ± 0.2210	0.4361 ± 0.1524	0.3883 ± 0.1153	**0.2836 ± 0.0934**	0.2922 ± 0.0726	0.8111 ± 0.2361	0.7408 ± 0.1913	0.8340 ± 0.2342
	$R^2$	0.5654 ± 0.3348	0.8962 ± 0.0565	0.9185 ± 0.0402	**0.9500 ± 0.0355**	0.9484 ± 0.0310	0.6687 ± 0.0904	0.6552 ± 0.2972	0.6323 ± 0.1351
$y_2$	RMSE	0.9581 ± 0.4406	0.9723 ± 0.4541	0.7302 ± 0.2493	**0.4041 ± 0.1262**	0.5490 ± 0.2599	0.5845 ± 0.1663	1.0670 ± 0.4693	0.7801 ± 0.2718
	$R^2$	0.7054 ± 0.2712	0.6943 ± 0.2890	0.8390 ± 0.1073	**0.9504 ± 0.0331**	0.9003 ± 0.0981	0.8991 ± 0.0578	0.6435 ± 0.3112	0.8214 ± 0.1173
$y_3$	RMSE	2.1621 ± 0.3605	0.9441 ± 0.2762	0.9043 ± 0.2632	0.6692 ± 0.2289	**0.6489 ± 0.1988**	1.8212 ± 0.6296	2.0846 ± 0.3685	2.2223 ± 0.6765
	$R^2$	0.4015 ± 0.3388	0.8786 ± 0.0687	0.8935 ± 0.0533	0.9325 ± 0.0515	**0.9349 ± 0.0469**	0.6138 ± 0.1897	0.4642 ± 0.2349	0.4430 ± 0.1812
$y_4$	RMSE	4.6375 ± 0.8277	4.3605 ± 0.8444	3.0487 ± 0.5676	2.1277 ± 0.5801	**2.0025 ± 0.6022**	4.1454 ± 1.2023	3.8531 ± 0.9148	4.8970 ± 0.9297
	$R^2$	0.8798 ± 0.0451	0.8935 ± 0.0464	0.9473 ± 0.0256	0.9744 ± 0.0129	**0.9755 ± 0.0192**	0.8942 ± 0.0862	0.9180 ± 0.0326	0.8639 ± 0.0619
$y_5$	RMSE	0.2897 ± 0.1139	0.2592 ± 0.1346	0.2729 ± 0.1131	**0.2132 ± 0.0973**	0.2403 ± 0.1231	0.2723 ± 0.0707	0.2780 ± 0.1095	0.2711 ± 0.1098
	$R^2$	0.3566 ± 0.6091	0.4985 ± 0.4599	0.4364 ± 0.5122	**0.5910 ± 0.3770**	0.4786 ± 0.5405	0.3436 ± 0.7390	0.4221 ± 0.4930	0.3952 ± 0.6394

Significant values are in [bold].

### Optimization results and experimental verification

For the prediction model *f* in the optimization problem, we used an ensemble of ten NN(10) models built during the cross-validation procedure. The ensemble typically yields more accurate and robust predictions than individual models by reducing the risk of overfitting^40^. The weight hyperparameters in the objective function $\tilde{{J}}$ were set based on the relative importance of individual objectives after discussion with domain experts. The hyperparameters $\alpha _2$, $\alpha _3$, $\alpha _4$ and $\alpha _5$ were all set to 10. The hyperparameters $\beta _1$ and $\beta _2$ were set to 0.001. To solve the optimization problem, the L-BFGS-B algorithm was implemented using the scipy package.

We optimized the operating conditions of the target system for seven example cases, each corresponding to a different initial state of the environmental variables $\textbf{z}^0$ (*i.e.*, the system environment), as listed in Table 4. For each case, the initial state of the decision variables $\textbf{x}^0$ (*i.e.*, the initial operating condition) was equally set to [3.0, 40.0] according to the manual operating condition used by domain experts. After the solution $\textbf{x}^*$ (*i.e.*, the optimized operating condition) was derived by solving the optimization problem for each case, we experimentally verified the system performance provided by the optimized operating conditions. Running the system under the optimized operating conditions, the actual values of the dependent variables $\textbf{y}$ (*i.e.*, system performance) were acquired to determine whether the objectives of the system operation were satisfied.

**Table 4:** Optimization and experimental verification results for the operation of the target system.

Case ID	Environmental variables ($\textbf{z}$)	State	Decision variables ($\textbf{x}$)	Predicted dependent variables ($\hat{\textbf{y}}$)	Actual dependent variables ($\textbf{y}$)
1	[3.2, 25.0, 43.0]	Initial	[3.0, 40.0]	$[25.7, {\textbf {97.7}}, -{\textbf {1.1}}, {\textbf {183.7}}, {\textbf {3.2}}]$	$[26.5, {\textbf {97.8}}, {\textbf {0.1}}, {\textbf {182.3}}, {\textbf {3.2}}]$
		Optimized	[2.0, 32.0]	$[{\textbf {24.0}}, {\textbf {98.3}}, -{\textbf {0.8}}, {\textbf {182.2}}, {\textbf {3.0}}]$	$[{\textbf {24.3}}, {\textbf {98.0}}, {\textbf {0.1}}, {\textbf {178.0}}, {\textbf {3.0}}]$
2	[7.2, 25.0, 43.0]	Initial	[3.0, 40.0]	$[25.4, {\textbf {97.9}}, -{\textbf {0.7}}, {\textbf {187.4}}, {\textbf {3.2}}]$	$[25.9, {\textbf {98.5}}, {\textbf {0.1}}, {\textbf {187.7}}, {\textbf {3.2}}]$
		Optimized	[2.0, 32.0]	$[{\textbf {23.7}}, {\textbf {98.2}}, -{\textbf {0.2}}, {\textbf {187.8}}, {\textbf {3.0}}]$	$[{\textbf {23.8}}, {\textbf {98.8}}, {\textbf {0.1}}, {\textbf {183.1}}, {\textbf {3.0}}]$
3	[10.8, 25.0, 43.0]	Initial	[3.0, 40.0]	$[25.1, {\textbf {97.0}}, -{\textbf {0.1}}, {\textbf {191.4}}, {\textbf {3.2}}]$	$[25.6, {\textbf {97.2}}, {\textbf {0.3}}, {\textbf {192.7}}, {\textbf {3.3}}]$
		Optimized	[2.0, 32.0]	$[{\textbf {23.4}}, {\textbf {97.4}}, {\textbf {0.6}}, {\textbf {194.0}}, {\textbf {3.0}}]$	$[{\textbf {23.5}}, {\textbf {98.4}}, {\textbf {0.5}}, {\textbf {189.7}}, {\textbf {3.1}}]$
4	[14.1, 25.0, 43.0]	Initial	[3.0, 40.0]	$[24.9, {\textbf {95.9}}, {\textbf {0.7}}, {\textbf {196.2}}, {\textbf {3.2}}]$	$[25.5, {\textbf {96.3}}, {\textbf {0.7}}, {\textbf {197.2}}, {\textbf {3.3}}]$
		Optimized	[2.1, 33.0]	$[{\textbf {23.2}}, {\textbf {96.3}}, {\textbf {1.3}}, {\textbf {200.4}}, {\textbf {2.9}}]$	$[{\textbf {23.0}}, {\textbf {97.4}}, {\textbf {1.0}}, {\textbf {197.7}}, {\textbf {3.1}}]$
5	[17.1, 25.0, 43.0]	Initial	[3.0, 40.0]	$[24.4, {\textbf {95.0}}, {\textbf {1.0}}, {\textbf {201.6}}, {\textbf {3.3}}]$	$[24.9, {\textbf {95.8}}, {\textbf {1.2}}, {\textbf {201.4}}, {\textbf {3.3}}]$
		Optimized	[2.2, 35.0]	$[{\textbf {23.0}}, {\textbf {95.4}}, {\textbf {1.9}}, {\textbf {206.3}}, {\textbf {3.0}}]$	$[{\textbf {22.8}}, {\textbf {97.9}}, {\textbf {1.6}}, {\textbf {199.5}}, {\textbf {3.1}}]$
6	[19.2, 25.0, 43.0]	Initial	[3.0, 40.0]	$[24.1, 94.3, {\textbf {1.3}}, {\textbf {205.7}}, {\textbf {3.3}}]$	$[24.6, 94.6, {\textbf {1.4}}, {\textbf {206.4}}, {\textbf {3.3}}]$
		Optimized	[2.3, 36.0]	$[{\textbf {22.8}}, 94.8, {\textbf {2.3}}, {\textbf {210.1}}, {\textbf {3.1}}]$	$[{\textbf {22.5}}, {\textbf {97.1}}, {\textbf {2.3}}, {\textbf {206.3}}, {\textbf {3.1}}]$
7	[21.1, 25.0, 43.0]	Initial	[3.0, 40.0]	$[23.9, 93.5, {\textbf {2.0}}, {\textbf {210.7}}, {\textbf {3.3}}]$	$[24.3, 94.1, {\textbf {2.0}}, {\textbf {212.2}}, {\textbf {3.4}}]$
		Optimized	[2.5, 37.0]	$[{\textbf {22.9}}, 93.8, {\textbf {2.5}}, {\textbf {214.9}}, {\textbf {3.1}}]$	$[{\textbf {23.1}}, {\textbf {95.9}}, {\textbf {1.7}}, {\textbf {207.6}}, {\textbf {3.5}}]$

Significant values are in [bold].

Table 4 lists the optimization and experimental verification results for the seven cases. For each case, we present the values of the decision variables, predicted dependent variables, and actual dependent variables in the initial and optimized states. The values of the dependent variables are shown in bold if their respective objectives were satisfied. Although the initial states of the decision variables were the same, the optimized states of the decision variables differed depending on the states of the environmental variables. The experimental verification results successfully demonstrated the effectiveness of the proposed method in improving the system performance. The optimized states always led to lower *energy consumption* ($y_1$) and higher *VOCs reduction rate* ($y_2$). Compared with the initial operating conditions, *energy consumption* ($y_1$) was reduced by 8.1% on average and the objectives for the other dependent variables ($y_2,\ldots ,y_5$) were all satisfied.

## Conclusion

We presented the data-driven autonomous operation method based on optimization and machine learning for CV-Master, a VOCs removal system developed by Shinsung E&G. We formulated an optimization problem by defining the decision, environmental, and dependent variables, identifying the constraints imposed on the decision variables, and designing the objective function representing the objectives of the system operation. Using past operational data of the system, a neural network was built to approximate the decision variables, thereby approximately representing the objective function as a function of the decision and environmental variables only. Given the current state of the system environment, the optimization problem was solved using the L-BFGS-B algorithm to derive the optimal values of the decision variables corresponding to the optimized operating conditions of the system. The experiments successfully demonstrated that the proposed method improved the operating conditions for the target system under various environmental states. Compared with the manual operating conditions used by domain experts, the operating conditions automatically derived by the proposed method reduced the energy consumption by 8.1% on average without violating any system constraints.

In practice, domain experts find it challenging to understand the relationship between the inputs and outputs of a complicated system. To circumvent this difficulty, the proposed method empirically learns the relationship from past operational data of the system in the form of a prediction model and uses the model to approximately express the objective function of the optimization problem. Consequently, the system can be operated in a data-driven manner without requiring an in-depth understanding of the mechanism of the system. In situations where the system environment changes over time, the proposed method allows the operating conditions of the system to be self-optimized without the need for manual intervention by domain experts, i.e., autonomous operation. Moreover, the scalability of the proposed method is not directly determined by the size and complexity of the target system, but rather depends on the number of decision variables, the types of objective function and constraints in the optimization problem, and the size and complexity of the neural network used as the prediction model. We believe that the proposed method can contribute to improving the autonomous operation of real-world industrial systems of various sizes and complexities.

An important consideration for the practical application of the proposed method is that the reliability of the optimization results significantly depends on the predictive performance of the prediction model used in the objective function. The prediction model exhibits poor predictive performance when the quantity and quality of training data is insufficient. The performance of the prediction model may also be degraded if the relationships between the variables in the target system change over time. We anticipate that this issue will be mitigated by using the predictive uncertainty of the prediction model as an indicator of the reliability of the proposed method. If the uncertainty at a certain moment is high, the operating conditions can be determined with the assistance of domain experts rather than relying solely on autonomous operation. Continuously updating the prediction model with new operational data will ensure better performance in the future.

## Data Availability

The dataset used in this study is available from the corresponding author on reasonable request.
